# Cross-cultural validation of Malay version of perceived professionalism among dental patients

**DOI:** 10.1038/s41405-024-00234-3

**Published:** 2024-06-07

**Authors:** Anitha Krishnan Pandarathodiyil, Shani Ann Mani, Suresh Kandagal Veerabhadrappa, Mahmoud Danaee, Ahmad Termizi Bin Zamzuri

**Affiliations:** 1 Faculty of Dentistry, SEGi University, No. 9 Jalan Teknologi, Kota Damansara, 47810 Petaling Jaya, Selangor Malaysia; 2 Department of Paediatric Dentistry and Orthodontics, Faculty of Dentistry, Universiti Malaya, 50603 Kuala Lumpur, Malaysia; 3 Department of Social and Preventive Medicine, Faculty of Medicine, Universiti Malaya, 50603 Kuala Lumpur, Malaysia

**Keywords:** Dental patient management, Dental treatments

## Abstract

**Background:**

Professionalism is a dynamic construct that requires constant revision based on contemporary practices and attitudes.

**Objectives:**

The purpose of this study was to cross-culturally adapt an already validated English questionnaire assessing patient perceptions of professionalism among dentists, into the Malay language.

**Methods:**

An original 24-item questionnaire was cross-cultural adapted in the Malaysian context through two phases. Phase I included content and face validity from experts’ evaluation which was followed by translation into the Malay language. Phase II involved psychometric assessment including construct validity and reliability analysis.

**Results:**

Expert evaluation indicated that all items demonstrated excellent content validity for the characteristics of relevance (CVI = 0.75–1.00 Kappa = 0.72–1.00) and clarity (CVI = 0.75–1.00 and Kappa= 0.72–1.00). A total of 300 dental patients completed the questionnaire. EFA was done on the first dataset and the second dataset was subjected to CFA which showed composite reliability (CR) ranging between 0.741 and 0.897 indicating acceptable reliability among items. The final questionnaire had 20 items with 3 domains; Patient expectation of a dental care provider, Ethics and Dentist’s professional responsibilities, Patient communication and confidentiality.

**Conclusion:**

This study has successfully validated the questionnaire for patient perception of professionalism in the Malaysian context.

## Introduction

The word “professionalism” is defined as “the skill, good judgment, and polite behavior that is expected from a person who is trained to do a job well” [1]. Extrapolating that definition to the world of healthcare, a dental professional today must adhere to an ‘unwritten’ set of rules of behavior, exhibit what is construed to be ‘good’ judgement (accepted by both the professional fraternity and the general public) and showcase a combination of hard and soft skills that result in patient satisfaction, goodwill, and effective treatment outcomes. A raft of studies has focused on patient perception of professional behavior by dental professionals [2–5]. All studies unanimously agree that the professional behaviour of a dental practitioner towards his/her patients is of critical importance but perceptions regarding professionalism of the health care provider varies between patients. The key traits expected by patients as regards professional behavior were ethical manner, good hygiene practices, honesty and confidentiality [6], while empathy was an important trait in another study [7]. Other studies addressed the patients perception of appropriate professional attire for the health care professional [8–10].

The twenty-first century comes with its own unique challenges, none more bewildering than the blazing technological revolution that leaves no sector unscathed in its wake. Armed with the advent of cutting-edge technology where any information hitherto considered sacrosanct could be gleaned by a few random clicks, the health care sector has had to contend with the paradigm shifts in patient needs and expectations, health care delivery systems, and strategies. William Osler opined that the practice of medicine is an art, not a trade, a calling, not a business—a calling in which your heart will be exercised equally with your head [11]. The field of dentistry is no different. Thus, it is of vital importance to define what is contemporary dental professionalism, how the dental professionals and patients perceive dental professionalism and most importantly; figure out how ‘convergent’ and ‘divergent’ their views are vis-à-vis dental professionalism.

Since what construes teaching of professionalism is not clear-cut and explicit, how do we teach/train our future dental professionals. Some opine that exposing students to literature helps to instill professional attributes while others aver that literature has no tangible benefits to offer on the professional front [6, 12]. Professionals in medical/dental education seem to concur with the idea that professionalism is a competency that can be achieved by measurable behaviors but these behaviors are ‘dynamic’ and thus, requires constant revision based on contemporary practices and attitudes. Therein lies the crux of the dilemma. The only way around in this scenario is to ensure continuous, evolving discussions with all the involved stakeholders (patient representatives, dental professionals, ethicists, dental associations, the government, and sociologists). As the adage goes ‘The customer is king’ and in the contemporary scenario, a patient-centered dental treatment/service is the need of the hour and needs to be considered while framing what is considered to be ideal/expected dental professional behaviour.

To this end, we decided to embark on a study to investigate the dental patients’ perception of ideal professional behavior of dentists and their dental team while providing quality dental care and describe what constitutes model dental professionalism based on these perceptions. We consider the patient’s perspective of what entails dental professionalism to be invaluable so that these perspectives can be inculcated into the curriculum and taught to the students as professional qualities that they can seek to pursue and master to become respected healthcare professionals in contemporary society. An already validated English questionnaire which was previous used in another country formed the basis of our study [4]. A similar study has not been conducted in Malaysia, to the best of our knowledge. The aim of this study was to cross-culturally adapt an already validated English questionnaire assessing patient perceptions of professionalism among dentists into the Malay language.

## Materials and Methods

The cross-cultural adaptation of a 24-item questionnaire on patients’ perception of professionalism in the Malaysian context included two phases. In Phase I, content and face validity were followed by translation into Malay language and in Phase II, the psychometric assessment including construct validity and reliability analysis were performed. The study was approved by the Medical Ethics Committee, Faculty of Dentistry, Universiti Malaya [DF OS2219/0046 (L)] and SEGi UC Ethics Committee, SEGi University [SEGiEC/StR/FOD/5/2022-2023].

### Research instrument

The questionnaire was adapted from the literature [4] which contained 24 items with four different domains, namely (1) excellence and communication skills; (2) humanism, commitment, and service-mindedness; (3) competence in practice; and (4) dentist’s duties and management skills. All items were assessed using a 5-point response scale consisting of totally agree, agree, undecided, disagree and totally disagree.

### Phase I

#### Face and content validity

In the present study, face and content validation was performed on items from the original questionnaire which was related to perception of professionalism among dental patients in Malaysia. Eight dental professionals (Table 1) with various specialist backgrounds, who were familiar with the terminology were invited to assess the relevance and clarity of items based on a four-point scale (1 = not relevant /clear, 2 = somewhat relevant /clear, 3 = quite relevant /clear, 4 = highly relevant/clear).

**Table 1:** Personal Details of Experts.

No.	Designation	Area of specialization	Place of Work	Nationality
1	Dental Specialist	Paediatric Dentistry	Universiti Malaya	Malaysia
2	Dental Specialist	Public Health	Universiti Malaya	Malaysia
3	Dental Specialist	Orthodontist	Universiti Sains Malaysia	India
4	Dental Specialist	Periodontist	SEGi University	India
5	Dental Specialist	Paediatric Dentistry	Universiti Teknologi Mara	Malaysia
6	Dental Specialist	Oral Surgery	Universiti Teknologi Mara	Malaysia
7	Dental Specialist	Oral Surgery	Manipal University College Malaysia (MUCM)	Malaysia
8	Dental Specialist	Orthodontist	MAHSA University	Malaysia

The feedback of expert’s evaluation was analyzed through calculation of the Content Validity Index (CVI) and Kappa coefficient. For each item, the CVI was calculated as the number of experts who provided a rank of 3 or 4, divided by the total number of experts. The Kappa Modified Coefficient was also used to determine the degree of agreement among experts [13]. The revised items and the comments on the content validity were deliberated amongst a core research team comprising of one paediatric dentist, public health dentist, oral pathologist, and periodontist each. The revision of the questionnaire was done based on the experts’ suggestions. The final questionnaire had 25 items which were subjected to the next phase of the study.

#### Translation to Malay language

The process of translation followed a published guideline [14], and involved several steps, including forward-translation from English to Malay, evaluation by the core research team, back- translation from Malay to English, re-evaluation by the core research team, and cognitive debriefing amongst a group of patients. Cognitive debriefing on potential participants was done to assess any difficulties in comprehension and the time taken to complete the questionnaire.

### Phase II : Psychometric assessment

The psychometric assessment including Exploratory Factor analysis (EFA), Confirmatory Factor analysis (CFA) and reliability of the Malay version of the questionnaire was done. The required sample size for both EFA and CFA was considered as 150 for each part [15]. Written informed consent was obtained from those who participated, and data was collected and tested for construct validity and reliability.

### Data collection

Data was obtained from Malaysians patients above 18 years of age, seeking dental treatment at two university dental clinics in Selangor and Kuala Lumpur, Malaysia (one private and one public) from April 2023 to June 2023. Patients who could speak and read the Malay language were invited to complete the questionnaire. Patients completed the paper-based questionnaire while waiting for dental treatment and after giving consent.

### Statistical analysis

The data collected was randomly divided into two parts. EFA was used to find the factor structure among 25 items on 140 samples. This was done by getting the Bartlett’s test of sphericity and the Kaiser-Mayer-Olkin measure of sample adequacy considering a KMO > 0.60 and a Bartlett’s significance level p < 0.05. To investigate the structure of the instrument, EFA using Promax rotation and Principal Axis Factoring approach was carried out. Additionally, parallel analysis was used to determine the number of extracted components. The reliability analysis for the components were examined using Cronbach’s Alpha coefficient. All analyses in this step were done using JASP version 0.17.1 (University of Amsterdam) and SPSS version 25 (IBM).

Based on the outcomes of EFA using SMART PLS Ver 4.0.9.5, the second portion of the data (160 samples) was used for confirmatory factor analysis (CFA) using CB-SEM (covariance-based SEM) to check the dimensionality of the factors. The measurement models were evaluated using the PLS-SEM approach, which also determined the convergent validity (0.50) and outer loading (>0.5) of the indicators. The reliability of the instrument was assessed using Cronbach’s alpha (>0.7) and composite reliability (>0.7). The HTMT technique (Hetrotrait-Monotrait ratio of criteria; 0.85) and cross loading values were used to test the discriminant validity.

## Results

### Phase 1

#### Content and Face validity

Based on the initial scrutiny of the questionnaire by the research team, one item (No.24) was disregarded since it was not applicable to the Malaysian population. The remaining 23 items of the questionnaire were sent to experts for content and face validity. Results of expert evaluation on both relevance and clarity for the items indicated that all items demonstrated excellent content validity for the characteristics of relevance (CVI = 0.75–1.00 Kappa = 0.72–1.00 and clarity (CVI = 0.75–1.00 and Kappa= 0.72–1.00) (Table 2). Items 1, 2, 3, 4, 8, 9, 17, and 22 were corrected according to the expert’s comments. Based on these results, all 23 items for this construct met the content and face validity. Two new items (Nos. 24 and 25) were added based on the feedback from the experts (Table 3).

**Table 2:** Relevancy and Agreement of the Instrument Items.

Item	Relevance		Clarity		Results
	CVI	kappa	CVI	kappa	
Q1	1.00	1.00	0.75	0.72	Validated
Q2	1.00	1.00	0.75	0.72	Validated
Q3	1.00	1.00	0.88	0.87	Validated
Q4	1.00	1.00	0.88	0.87	Validated
Q5	1.00	1.00	1.00	1.00	Validated
Q6	1.00	1.00	1.00	1.00	Validated
Q7	1.00	1.00	1.00	1.00	Validated
Q8	1.00	1.00	0.88	0.87	Validated
Q9	1.00	1.00	1.00	1.00	Validated
Q10	0.88	0.87	0.88	0.87	Validated
Q11	1.00	1.00	1.00	1.00	Validated
Q12	1.00	1.00	0.88	0.87	Validated
Q13	0.88	0.87	0.88	0.87	Validated
Q14	1.00	1.00	0.88	0.87	Validated
Q15	1.00	1.00	1.00	1.00	Validated
Q16	0.75	0.72	1.00	1.00	Validated
Q17	1.00	1.00	0.88	0.87	Validated
Q18	1.00	1.00	1.00	1.00	Validated
Q19	1.00	1.00	0.88	0.87	Validated
Q20	1.00	1.00	1.00	1.00	Validated
Q21	0.88	0.87	1.00	1.00	Validated
Q22	0.88	0.87	1.00	1.00	Validated
Q23	1.00	1.00	1.00	1.00	Validated

**Table 3:** Patient’s perception of professionalism among dentists.

*No*.	*Item*
1.	Ethical decisions by the dental team are very important for patient care that is ethically good and maintaining suitable personal behaviour at all times (honest and fair), ensures that patients and colleagues have trust and faith confidence in them.
2.	Members of the dental team must adhere to the regulations and procedures of sterilisation and antisepsis, as well as follow the standards and guidelines for good dental practice.
3.	Members of the dental team should act in a way that shows respect for the patient and his family.
4.	Good personal hygiene and neat grooming are important.
5.	The dentist must document data and patient treatment accurately.
6.	The dentist should use the best clinical and diagnostic considerations to avoid any mistakes in the patient’s dental care while giving the most efficient dental care.
7.	Members of the dental team should respect patients’ rights to make their own decisions regarding their own treatment (when the patient is able to make a decision).
8.	Members of the dental team must take responsibility for all decisions regarding patient care.
9.	Members of the dental team must work in a collaborative manner with other medical professionals for the good of the patient.
10.	It is acceptable to release patient’s dental information to family members.
11.	It is the responsibility of the members of the dental team to use their knowledge and skills to assist and offer dental care to everyone.
12.	Members of the dental team should seek and maintain a high standard of specialisation in both dental practice and patient care.
13.	Generally, patients are influenced by actions (all body language except words) of the dental team.
14.	Members of the dental team should have a positive caring attitude.
15.	The quality of the dental care depends on punctuality, organised time management in the clinic
16.	It is acceptable to share patient information with other patients.
17.	A dentist should explain to the patient his condition and all treatment options and cost-effective treatments in a way that is easy to understand and check the patient’s understanding of the treatment plan.
18.	Members of the dental team should be aware of their responsibility to improve the dental health and knowledge of the public.
19.	Members of the dental team should weigh the importance of the patient over the importance to themselves.
20.	Members of the dental team should maintain good communication with all patients, colleagues and other health providers.
21.	Patients should be aware of their legal rights and responsibilities that govern the doctor/patient relationship.
22.	As long as the members of the dental team carry out the correct treatment/care, there is no need to talk and explain to the patient.
23.	It is the patient’s responsibility to report any information regarding illegal practice or unethical practice of the dental team.
24.	The dental team can make videos of dental clinic events that involve patients and share them on social media without their consent.
25.	It is important for a dental clinic to have a platform for patients’ complaints and feedback to improve the service.

#### Translation and Cognitive de-briefing

The translation of the instrument was conducted as per the methods stated below. The forward-translation was done by two native Malay- speaking translators: one paediatric dentist, and one public health dentist. The core research team, which included one paediatric dentist, public health dentist, oral pathologist and periodontist each, evaluated these two forward-translated versions before achieving a consensus version.

The Malay consensus version of the questionnaire was then back-translated into English by two independent English-speaking translators who were also proficient in the Malay language: both were specialists in public dental health and different from the team involved in forward-translation. Back-translated English version was then compared to the original English language to achieve conceptual and semantic equivalency. Following the same core research team’s recommendations, the Malay version of the questionnaire was finalized. Cognitive debriefing was done on fifteen patients attending two university dental clinics, one private and one public. On average, participants took 5–6 minutes to complete the questionnaire.

### Phase II (construct validity)

#### Sociodemographic characteristics

A total of 301 dental patients completed the questionnaire. Regarding sociodemographic characteristics, more respondents were female and in the 18-24 years age group and were Chinese and single (Table 4).

**Table 4:** Sociodemographic characteristics of respondents for EFA and CFA Sample.

Variable	Level	EFA (*n* = 140)	CFA (*n* = 161)
**Gender**	Male	51(36.4)	66(41)
	Female	89(63.6)	95(59)
**Age**	18–24	62(44.3)	85(52.8)
	25–34	35(25)	39(24.2)
	35–44	13(9.3)	9(5.6)
	45–54	13(9.3)	17(10.6)
	55–64	11(7.9)	8(5)
	65 and above	6(4.3)	3(1.9)
**Race**	Malay	60(42.9)	46(28.6)
	Chinese	64(45.7)	85(52.8)
	Indian	10(7.1)	18(11.2)
	Others	6(4.3)	11(6.8)
**Marital Status**	Married	46(32.9)	41(25.4)
	Single	92(65.7)	116(72)
	Divorced	0(0)	1(0.6)
	Widowed	2(1.4)	3(1.9)
**Total Household Income**	< RM5000	84(60)	83(51.6)
	RM5000–10,000	42(30)	53(32.9)
	>RM 10,000	14(10)	25(15.5)
**Highest level of Education**	No formal education	2(1.4)	3(1.9)
	Primary school	7(5)	2(1.2)
	Secondary school	26(18.6)	30(18.6)
	Diploma	28(20)	30(18.6)
	Degree	63(45)	83(51.6)
	Postgraduate	14(10)	13(8.1)
**Visited dentist/received dental treatment before**	No	2(1.4)	1(0.6)
	Yes	138(98.6)	160(99.4)
**Frequency of receiving dental treatment**	Once in 2-3 years	14(10)	28(17.4)
	Whenever I have dental problems	62(44.3)	65(40.4)
	Once a year	33(23.6)	35(21.7)
	Twice or more in a year	31(22.1)	33(20.5)

#### Exploratory factor analysis (EFA)

**Fig. 1:**
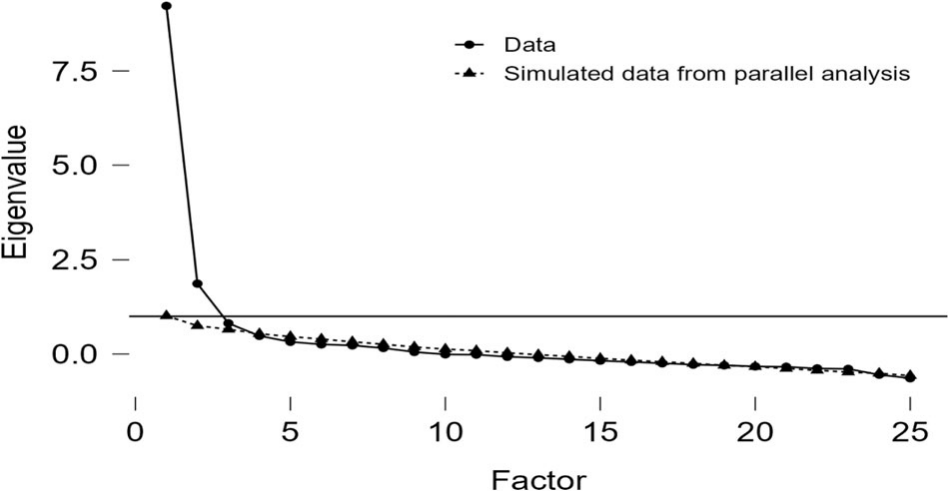
Scree plot and parallel analysis. Scree plot of Eigenvalues from the factor analysis and results of the parallel analysis on simulated data underlying 25 items.

Exploratory Factor Analysis using Promax rotation was applied to determine the factor structure among 25 items related to patient’s perception of professionalism among dentists (Table 5). The KMO test assesses the sampling adequacy for factor analysis. The overall KMO value obtained was 0.903, indicating a highly suitable dataset for factor analysis. Individual item KMO values ranged from 0.676 to 0.952, all demonstrating excellent suitability. Bartlett’s test examines whether the correlation matrix is significantly different from the identity matrix and results showed that the Bartlett’s test of sphericity was significant (χ^2^ = 2003.257, *p* < 0.05). In the current study, all initial communalities were above the threshold. The results of EFA on all 25 items extracted three components based on parallel analysis (Fig. 1). Three items were removed due to cross loading (Q14), negative loading (Q23) and low loading factor (Q8) from the questionnaire in this phase. The final results revealed that the first component is related to *Ethics and Dentist’s professional responsibilities* with 7 items and explained 22.2% of the variance. The second factor including 12 items (*Patient expectation of a dental care provider)* explained 20.7% of the variance. The third component included three items regarding *Patient communication and confidentiality* which explained 7.4% of the variance. Total variance explained by these three components was 50.2% which was greater than the recommended value of 50% as a general rule (Streiner, 1994). Results of the reliability analysis using alpha Cronbach showed (range 0.768 to 0.913) that all the factors had an alpha value greater than standard of 0.70.

**Table 5:** Factor loadings based on promax rotation for 25 items related to patient perception of professionalism among dentists.

Item	Factor 1	Factor 2	Factor 3
Q4	1		
Q6	0.944		
Q3	0.911		
Q5	0.818		
Q2	0.74		
Q1	0.535		
Q17	0.489		
Q14^a^	0.445	0.457	
Q20		0.744	
Q11		0.723	
Q21		0.668	
Q19		0.659	
Q18		0.597	
Q12		0.596	
Q13		0.589	
Q7		0.512	
Q10		0.502	
Q25		0.431	
Q9		0.430	
Q15		0.423	
Q8^b^		0.323	
Q23^c^		-0.250	
Q24			0.886
Q22			0.724
Q16			0.646
Eigenvalues	5.544	5.165	1.852
% of Variance	22.2	20.7	7.4
alpha	0.913	0.881	0.768

^a^Removed due to cross loading, ^b^Removed due to low loading ^c^Removed items due to negative loading.

#### Confirmatory Factor Analysis (CFA)

**Fig. 2:**
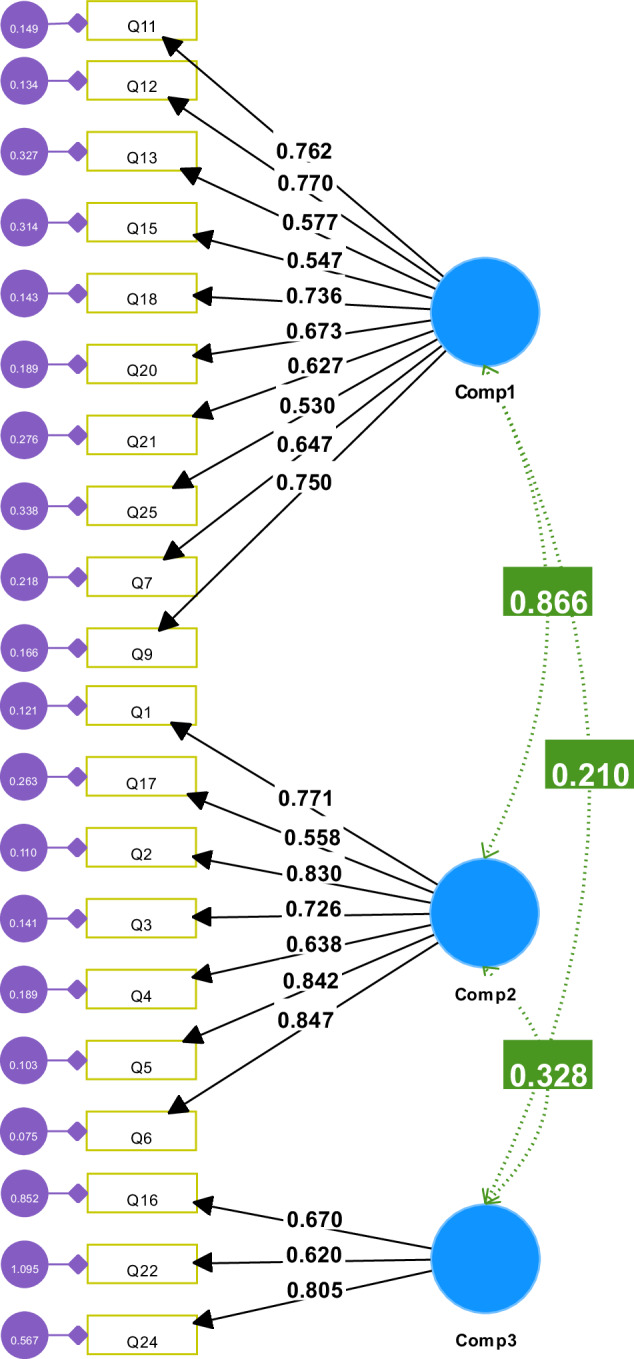
Modified measurement model: Confirmatory factor analysis. Note. χ^2^/df = 1.99, *p* ≤ 0.001; CFI = 0.90, RMSEA = 0.079, SRMR = 0.06. Standardized path estimates shown. All loading paths and covariances are significant at *p* < 0.05.

Based on the results of EFA in the first data set, confirmatory factor analysis was conducted on second data set (*n* = 161) using Smart-PLS Ver 4 (Fig. 2). The results of initial model indicated lack of fit despite the acceptable level for χ^2^/DF = 2.002, GFI = 0.816 and SRMR = 0.066, RMSE = 0.079, while CFI = .88 was below the cut-off point of 0.9. It was found all items loading were above the threshold (0.5) except two items including Q10 and Q19 which were removed in modified model. The measurement model results for all constructs after modification showed that all items had a loading above 0.5 which were above the threshold (0.5) and indicated that modified model was fitted with χ^2^/DF = 1.99, GFI = 0.84; CFI = 0.90 and SRMR = 0.06. In addition, the RMSEA = 0.079 met the cut-off point 0.08, which was between the recommended range of acceptability. The modified measurement model reveals lower AIC (ΔAIC = −87.357) and BIC (ΔBIC = −99.683), demonstrating substantial improvement in modified model fit compared to the initial model. According to the result of the current study composite reliability (CR) ranged between 0.741 and 0.894 indicating acceptable reliability among items. In addition, in this study, average variance extracted values revealed that these three components had an adequate convergent validity (AVE > 0.4) (Table 6). The results of the reliability analysis revealed Cronbach’s alpha values ranging from 0.737 to 0.894, all of which exceeded the commonly accepted threshold of 0.7.

**Table 6:** The result of Convergent Validity and reliability of measurement model.

Construct	Item	Outer loadings (standardized)		Cronbach’s alpha	CR	AVE
		Initial model	Modified model			
**Component 1**	Q11	0.767	0.762	0.888	0.883	0.446
	Q12	0.761	0.77			
	Q13	0.581	0.577			
	Q15	0.554	0.547			
	Q18	0.741	0.736			
	Q19	0.425	Deleted			
	Q7	0.647	0.646			
	Q9	0.742	0.75			
	Q20	0.676	0.673			
	Q21	0.64	0.627			
	Q25	0.53	0.53			
**Component 2**	Q17	0.558	0.558	0.894	0.897	0.565
	Q2	0.83	0.83			
	Q3	0.728	0.726			
	Q4	0.637	0.638			
	Q5	0.841	0.842			
	Q6	0.847	0.847			
	Q1	0.77	0.771			
**Component 3**	Q16	0.672	0.67	0.737	0.741	0.494
	Q22	0.621	0.62			
	Q24	0.802	0.805			

Heterotrait-Monotrait ratio (HTMT) of correlations was used to assess discriminant validity [16]. Table 7 displays HTMT values for each construct in this investigation. The constructs have discriminant validity as all values were < 0.9.

**Table 7:** The result of Discriminant Validity using HTMT method.

	Component 1	Component 2	Component 3
**Component 1**	–		
**Component 2**	0.867	–	
**Component 3**	0.243	0.337	–

## Discussion

The study aimed to validate the Malay version of the Perceived Professionalism questionnaire for dental patients. The final questionnaire had 20 items and underwent translation to the Malay language. Maintaining the validity and reliability of instruments and scales in questionnaire translation is crucial for cross-cultural adaptation [17]. The literature has proposed a six-step model for cross-cultural adaptation of an index [18]. These steps include ensuring conceptual, item, semantic, operational, and measurement equivalence, which collectively enhance the validity and reliability of assessments in diverse cultural contexts. Achieving functional equivalence requires success in all types of equivalence [19, 20].

The meticulous evaluation of the questionnaire by the research team, leading to the exclusion of item no. 24 due to its inapplicability to the Malaysian population. Item no. 24 of the original questionnaire displayed male dentists in different clothing, requiring respondents to select an image of a dentist they would prefer receiving treatment from. Given that Malaysia is a melting pot of three diverse cultures [21, 22] Malay, Chinese, and Indian, the definition of standard professional attire can vary significantly. Moreover, with the proportion of female dentists nearly doubling that of male dentists in our country, the item could potentially reflect a gender bias, rendering it unsuitable for inclusion. Owing to the challenges associated with representing all appropriate attires in visual form within the questionnaire, we made the decision to eliminate this item. This highlights the importance of contextualizing assessment tools for different cultural settings. This decision was in line with best practices in cross-cultural research, ensuring that the instrument is culturally sensitive and meaningful.

In our study, the high CVI scores, ranging from 0.75 to 1.00, along with robust Kappa values (0.72–1.00), suggested that all retained items possess excellent content validity for both relevance and clarity [23, 24]. The correction of certain items (1, 2, 3, 4, 8, 9, 17, and 22) and incorporation of two new items (No. 24 and 25) based on expert feedback was a notable aspect of the face validity assessment and ensures that the questionnaire aligns with the cultural nuances that may have been overlooked in the original version and specifics of the dental profession in Malaysia. The new item no. 24 was included to gauge patients’ opinions on whether the dental team should be permitted to create and post videos of clinic events involving patients on social media without their explicit consent, considering the widespread reliance of the public on social media for information and the use of social media by dentists for marketing [25]. Additionally, the new item no.25 was aimed to investigate if patients felt it was pivotal to be provided a channel for complaints and feedback at dental clinics to enhance service quality [26]. By assessing their perspectives, the data could then be used to potentially enhance dental services for upcoming patients as well as for themselves seeking treatment in the future.

Technical equivalence refers to data collection methods and procedures being comparable across languages and cultures using the same tool [27]. The process involved forward-translation, evaluation by an expert committee, back-translation, re-evaluation, and pre-testing among patients [27]. Forward translation preserves the original questionnaire’s meaning and intent, while back translation involves a different translator to identify discrepancies. In our study, two native Malay-speaking translators completed the forward translation: a public health dentist and a paediatric dentist. Before reaching a consensus version, the core research team evaluated these two forward-translated versions. Two independent English-speaking translators, who were experts in public oral health and also fluent in Malay, back-translated the questionnaire from the consensus version into English. Back-translated English version was then contrasted with the source language to establish conceptual and semantic comparability. The Malay version of the questionnaire was completed in accordance with the suggestions made by the core research team.

Validation involves testing the questionnaire in the target culture, including Cognitive de-briefing, statistical analysis, and expert review [28]. The Malay version was finalized and pre-tested on fifteen patients at two university dental clinics. There were no difficulties in comprehension during the pre-testing of the questionnaire. This underscores the precision and appropriateness of the translated items in capturing the intended construct of perceived professionalism among dental patients within the Malaysian cultural context.

The sociodemographic characteristics of respondents provide valuable insights into the diversity of the sample. The predominance of female respondents in both the EFA and CFA samples reflects the common trend of higher female representation in healthcare-related studies, which aligns with the demographics of Malaysian dental patient populations [29]. The age group of 18-24 constitutes the largest proportion in both samples. This skew towards a younger age group may reflect the overall demographics of dental patients or suggest a greater willingness among younger individuals to participate in research. This finding was in accordance with a study by Tan, YR et al., who reported demographic and socioeconomic inequalities in oral healthcare utilization by Malaysians [29]. The Chinese ethnic group was prominently represented in both samples, followed by the Malay group. Understanding the perceptions of different ethnic groups is crucial for the cross-cultural validation of the perceived professionalism questionnaire, as cultural nuances may influence responses [30].

The majority of respondents in both samples were single. This distribution may have implications for the perceived professionalism construct, as marital status can influence healthcare-seeking behavior and expectations from healthcare providers. A significant proportion of respondents in both samples fall within the <RM5000 income bracket. This socioeconomic diversity was relevant in assessing whether perceptions of professionalism vary across different economic strata. A notable proportion holding a degree in both samples suggests the influence of health literacy and, consequently, perceptions of professionalism towards dentists. The overwhelming majority of respondents had visited a dentist or received dental treatment before. The high level of previous dental experience ensures that the perceptions captured in the questionnaire are grounded in real-life encounters with regard to dental professionals [31].

The implementation of EFA yielded meaningful insights into the factor structure and underlying components influencing patient perceptions. The KMO value of 0.903 indicates a highly suitable dataset for factor analysis, emphasizing the adequacy of the sample for the examination of factor structures. The significance of Bartlett’s test further supports the appropriateness of applying factor analysis to the dataset, given the correlation matrix’s departure from the identity matrix [32].^.^

The EFA revealed that the items were grouped into three distinct components; *Patient expectation of a dental care provider, Ethics and Dentist’s professional responsibilities, and Patient communication and confidentiality*. Under the component of ‘*patient communication and confidentiality’*, two new items pertaining to posting videos in social media without patient consent and an avenue for obtaining feedback and complaints. The factor structure was slightly different from the original questionnaire which had four factors; namely Excellence and communication skills, competence to practice, humanism and service mindedness and dentists duties and management skills [4]. The diverse ethnic composition of Malaysia contributes to the differing perspectives through which patients may interpret the contents of a questionnaire. Each cultural group, such as Malay, Chinese, Indian, and indigenous communities, holds unique values, beliefs, and norms that impact their views on professional behavior, communication styles, preferences, and healthcare practices. For instance, different ethnicities may either place varying levels of importance on a dentist’s authority and expertise, or on their warm and friendly demeanor. Moreover, specific cultural beliefs about oral health can also affect patients’ expectations and perceptions of dental care. Recognizing and understanding these cultural nuances are essential for evaluating how patients assess the professionalism of dental practitioners in Malaysia.

Three items were removed in this study due to cross-loading (Q14), negative loading (Q23), and low loading factor (Q8). The same items had lower ranking in the list of important elements of dental professionalism in the original study [4] Similar themes of perceived professionalism by patients were noted in other studies [2, 5]. The factor loadings for items exhibit substantial loadings, indicating their strong association with the respective components [33]. The Eigenvalues and the percentage of variance explained by each factor provide additional clarity on the relative importance of each component in explaining the overall perceived professionalism among dental patients. Reliability analysis using alpha Cronbach coefficients demonstrated strong internal consistency for all three factors, with values ranging from 0.768 to 0.913, exceeding the standard threshold of 0.70 suggesting that the retained items within each factor reliably measure the underlying constructs of professionalism [34].

The CFA conducted in the second dataset (*n* = 160) to validate the factor structure identified through the EFA in the initial dataset. The modified model demonstrated a good fit, as indicated by the χ2/DF ratio of 1.99. The values of other fit indices, such as the Goodness of Fit Index (GFI = 0.84), Comparative Fit Index (CFI = 0.90), and Standardized Root Mean Square Residual (SRMR = 0.06), also suggest an acceptable fit. The Root Mean Square Error of Approximation (RMSEA), falling within the recommended range (0.08), further supports the model’s adequacy. The measurement model indicated that, after modification, all items had standardized loadings above 0.5, except for Q19, which was subsequently removed in the final model. This suggests that most items reliably measure their respective constructs within the modified model, meeting the conventional threshold for satisfactory item loading. The assessment of reliability using CR demonstrated acceptable internal consistency among items for each component. The range of CR values (0.741 to 0.897) indicates robust reliability within each construct.

Convergent validity, assessed by Average Variance Extracted (AVE), indicated that all three components reliably capture a significant amount of variance from their respective sets of items. The standardized loadings for items within the component 1: *Patient expectation of a dental care provider* (Q11, Q12, Q13, Q15, Q18, Q19, Q7, Q9, Q20, Q21, Q25) all surpassed the acceptable threshold of 0.5, except for Q19, which was removed due to its lower loading factor. The reliability and convergent validity of this component were well-established. The items within component 2: *Ethics and Dentist’s professional responsibilities* (Q17, Q2, Q3, Q4, Q5, Q6, Q1) exhibited standardized loadings exceeding 0.5, demonstrating their reliability and contribution to the underlying construct. The reliability and convergent validity for Component 2 were notably strong. Lastly, the items in component 3: *Patient communication and confidentiality* (Q16, Q22, Q24) demonstrated acceptable standardized loadings, reliability, and convergent validity, contributing to the overall construct.

The HTMT ratio of correlations was employed to evaluate the discriminant validity to ensure that the constructs measured by the instrument were distinct from each other. The HTMT values for Component 1, 2, and 3 were below the threshold of 0.9, indicating satisfactory discriminant validity [16]. The HTMT values of 0.867 between Component 1 and Component 2, and 0.337 between Component 2 and Component 3, fall below the recommended threshold, signifying that these constructs are distinct from each other. The successful demonstration of discriminant validity provides assurance that the Malay version of the questionnaire effectively captures distinct facets of professionalism among dental patients. It is crucial for the instrument to differentiate between these components, as it ensures that the factors measured are not overlapping and contribute uniquely to the overall construct of perceived professionalism.

The modified measurement model is a refined version based on the outcomes of the CFA. This model incorporates feedback from the exploratory phase, displays a satisfactory fit with the data. The fit indices, such as χ2/DF, GFI, CFI, SRMR, and RMSEA, collectively indicate an acceptable fit of the model.

This study is not without limitations. Firstly, the gender representation in the sample population is imbalanced and the age range is not wide enough to represent the entire Malaysian population. Secondly, comparative analysis between the original 4-factor model and the proposed 3-factor model could not be done. Such an examination would afford a comprehensive discussion on the cultural disparities inherent in the questionnaires.

## Conclusion

In conclusion, the study’s rigorous methodology in developing and validating the questionnaire provides valuable insights into patients’ perceptions of professionalism in dental care. However, future research should consider the potential biases in patient feedback and aim for cross-cultural validation to enhance the applicability of the findings in diverse healthcare settings.

## Data Availability

The data that support the findings of this study are available from the corresponding author, [SAM], upon reasonable request.
